# Report from ‘ESCP 2021 Virtual’: the 16th Scientific and Annual Conference of the European Society of Coloproctology, 22–24 September 2021

**DOI:** 10.1111/codi.16061

**Published:** 2022-02-07

**Authors:** Miguel Cunha, Zoe Garoufalia, Vittoria Bellato, Yongbo An, Nagendra N. Dudi‐Venkata, Cristián Gallardo, Gloria Zaffaroni, Erman Aytac, Richard R. W. Brady, Gianluca Pellino

**Affiliations:** ^1^ ESCP Social Media Committee Co‐Chair Portsmouth UK; ^2^ Surgical Department Algarve University Hospital Portimão Portugal; ^3^ 219819 Department of Colorectal Surgery Cleveland Clinic Florida Weston Florida USA; ^4^ Second Propaedeutic Surgery Clinic Laiko University Hospital National and Kapodistrian University of Athens Athens Greece; ^5^ Minimally Invasive Surgery Unit Universitò di Tor Vergata Rome Italy; ^6^ Gastroenterology Surgery Department San Raffaele Hospital Milan Italy; ^7^ Beijing Friendship Hospital Capital Medical University Beijing China; ^8^ 1062 Colorectal Unit Department of Surgery Royal Adelaide Hospital Adelaide South Australia Australia; ^9^ Faculty of Health and Medical Sciences Adelaide Medical School University of Adelaide Adelaide South Australia Australia; ^10^ Servicio de Coloproctologia Hospital Clínico San Borja Arriarán Santiago Chile; ^11^ Department of General Surgery Hospital L. Sacco Milan Italy; ^12^ Department of Surgery School of Medicine Atakent Hospital Acibadem Mehmet Ali Aydinlar University Istanbul Turkey; ^13^ Incoming ESCP Communication Committee Chair Portsmouth UK; ^14^ Newcastle Centre for Bowel Disease Research Group Newcastle upon Tyne Hospitals NHS Foundation Trust and Newcastle University Newcastle upon Tyne UK; ^15^ ESCP Communication Committee Chair Portsmouth UK; ^16^ Department of Advanced Medical and Surgical Sciences Universitá degli Studi della Campania ‘Luigi Vanvitelli’ Naples Italy; ^17^ Colorectal Surgery Vall d’Hebron University Hospital Barcelona Spain

Following the unprecedented nature of the European Society of Coloproctology’s (ESCP’s) Virtually Vilnius in 2020 (the first completely online annual conference for the ESCP), the ESCP executive had hoped for better times. With news of vaccines and Covid infection rates reducing across Europe, there was considerable hope that 2021 would bring a much sought, and renewed, opportunity for a face‐to‐face congress. However, despite earlier optimism, a resurgence of Covid variant infections across Europe through mid‐2021 forced the Executive to again make the urgent and difficult decision to opt to broadcast our annual congress virtually, cancelling detailed plans for the face‐to‐face component in Barcelona in September.

Considerable human effort, across all the Society’s committees, was mobilized to deliver ESCP 2021 Virtual, to be broadcast in the form of a 3‐day virtual event. The executive was delighted with the resulting engagement, with more than 1400 participants from 80 countries attending between 22 and 24 September. Since then, the online recordings of the sessions have been viewed more than 12,000 times. The conference programme was an expert balance of international speakers and chairs, including several young ESCP members, and allowed open interaction and discussion of cases.

The congress was opened by the ESCP President, E. Xynos (Greece), who welcomed the virtual attendees and reported on the activities and initiatives of the Society, delivery of which continued despite the challenges caused by the pandemic.

E. Angenete (Sweden) was the first speaker on the virtual stage, giving a keynote lecture on the epidemiology of colorectal cancer (CRC). She highlighted the increasing incidence and severity of presentation of CRC among young adults but also that they have a higher disease‐free survival if treated adequately and in a timely manner. She linked her presentation to the challenge of the United Nation’s ‘sustainable development goals’ to reduce by a third the premature mortality associated with noncommunicable diseases by 2030. She advocated that we address this through better CRC care, screening implementation programmes and research. Notably, the sustainability agenda in surgery has been a major online focus for the ESCP this year.

The highly successful session on guidelines was moderated by J. Warusavitarne (UK). S. Breukink (Netherlands) introduced the ESCP guidelines for haemorrhoidal disease [1], with 34 recommendations covering six areas (evaluation of symptoms, diagnosis and classification, basic treatment, outpatient procedures, surgical interventions, special situations and other surgical techniques). E. Angenete presented the diverticulitis guidelines [2]: indications for emergency surgery, surgical approach, primary anastomosis and other important aspects were discussed. Y. Maeda (UK) reported on the guidelines for the diagnosis and treatment of faecal incontinence, as a collaboration between United European Gastroenterology, the ESCP, the European Society of Neurogastroenterology and Motility and the European Society for Primary Care Gastroenterology, emphasizing the importance of involving patients in shared decision‐making throughout the treatment of faecal incontinence. Complex case discussions were also included in this session.

M. Pera (Spain) introduced the ‘Surviving CRC’ session. C. Molenaar (Netherlands) stressed that most European countries lack a uniform definition of interval times (time periods since the diagnosis, the correct staging and the multidisciplinary final decision) for CRC pathways, and that recommendations on treatment intervals are often conflicting. K. Emmertsen (Denmark) discussed long‐term morbidity after CRC surgery, arguing that dedicated screening and treatment programmes can identify several patients with postsurgical sequelae (20%–30% of patients at her centre).

S. Ng (Hong Kong, China), co‐chair of the Global Reach Committee, opened the ‘Building and developing the global reach community’ session, with a global panel. D. Morton (UK) reported on the status of the ESCP EAGLE trial, followed by S. Mantoo (Singapore) who talked on the East of DAMASCUS trial, aimed at determining international variability in management of acute colonic diverticulitis. A. Banghu (UK) reported on the lessons learned with the CovidSurg studies, demonstrating a highly effective global collaboration producing rapid and clinically relevant data to guide decisions during a pandemic. N. Smart (UK) and S. Blackwell introduced the PROPHER (Patient Reported Outcomes after Parastomal HErnia tReatment) study, which will use advanced methods of collecting patients’ reported data. The panel discussion focused on study design, methodology and study participating requirements and inclusion criteria.

The joint ESCP–European Hereditary Tumour Group (EHTG) ‘Picking the right operation for the patient’ was chaired by G. Möslein (Germany) and A. Latchford (UK), who emphasized the importance of ‘dynamic guidelines’. A. Lepistö (Finland) presented an update on the guidelines for Peutz–Jeghers syndrome [3], noting that baseline screening for polyps should be performed at the age of 8 years. Treatment of intussusception was addressed in detail (conservative therapy, resolution of intussusception and resection of the pedunculated polyp). G. Zaffaroni (Italy–Germany) presented a preview of the EHTG–ESCP update guidelines for familial adenomatous polyposis (FAP), emphasizing the importance of considering FAP and attenuated FAP as different entities, and the potential role of minimally invasive surgery to reduce infertility rates and the development of desmoids.

The European Society of Gastrointestinal Endoscopy session ‘Management of bowel endometriosis’, chaired by S. Becker (Germany), included a multidisciplinary panel of experts representing the complex nature of managing this condition with access to expertise across specialities. The panel comprised M. Nisolle (Belgium), H. Ferreira (Portugal) and J. J. Tuech (France). Bowel‐sparing approaches are to be favoured where possible, but full‐thickness and mucosal involvement are indications for resection and rectal resection requires cross‐speciality engagement.

D. Hahnloser (Switzerland) chaired the ‘My most challenging case’ educational session, a series of difficult cases presented by trainees and commented on by senior surgeons. V. Laurenti (Italy) and A. Spinelli (Italy) presented a patient with recurrent Crohn’s disease (CD) with multiple enterocutaneous fistulas that required a carefully perioperative optimization followed by meticulous surgical planning; P Curchod and D. Clerc (Switzerland) showed two difficult cases of acute appendicitis, focusing on the pros and cons of conservative and operative management and discussing interval appendicectomy; T. Gregoir and M. Potter (UK) brought two different situations for which Hartmann resection was required; lastly, S. Stefan (UK) and A. Senapati (UK) presented a tailored approach to complex fistula‐in‐ano associated with perianal sepsis. The session was followed by a question and answer session with animated participation of virtual attendees.

W. Bemelman (Netherlands) chaired the ‘New techniques for fistula‐in‐ano: do they hold promise?’ session. S. Breukink (Netherlands) suggested that it is difficult to reach conclusions on the use of platelet‐rich plasma therapy, and indications should be further researched. P. Tozer (UK) provided some technical tips for video‐assisted anal fistula treatment, and showed the potential aims of the treatment: cure, palliation and morphological change. The success rates may be lower than previously assumed, but as a symptom‐palliation technique it represents is a good addition to the surgeon’s armamentarium. A. Rojanasakul (Thailand) emphasized the utility of ligation of the intersphincteric fistula tract (LIFT) even for fistulas with abscess, recalling that postoperative infection remains the biggest risk factor for recurrence, and provided tips to optimize the outcomes. S. Riss (Austria) reported on stem cell therapy, with a particular focus on the results obtained in patients with CD: a multidisciplinary approach and patient selection are essential.

The second day opened with the international trials results forum, reporting on early, unpublished results from the most recent worldwide trials. During this session, results were disseminated to the audience from the FALCON trial [4] by A. Banghu (UK), the ALASSCA trial (NCT02647099) by A. Martling (Sweden), LASER [5] by V. Sallinen (Finland), the CAPACITY1 trial [6] by C. Norton (UK), DAMASCUS by D. Vimalachandran (UK) and COMPASS by W. Xu (New Zealand). The session was chaired by D. Morton and C. Buskens (Netherlands), who promoted a fruitful discussion.

R. Brady (UK) introduced the keynote lecture by G. Pellino (Italy) that provided an overview of social media (SoMe). He argued that the academic and scientific value of SoMe has been demonstrated, but there is a need now for importance of ‘netiquette’, attention to detail and responsible actions, and a need for conscious development of ‘meta‐ethics’. Later, in another keynote, K. Matzel (Germany) introduced ‘Training must be more than an (interesting) educational experience’ by A. Gallagher (Belgium). During an impressive talk, he showed the importance of training within a training pathway and focused on metric‐based training as part of a proficiency‐based progression.

A. Spinelli (Italy) chaired the session on acute severe ulcerative colitis (UC). T. Raine (UK) spoke about current medical treatment for patients with acute severe UC. Despite current guidelines, the challenges of medical treatment are prompt identification of patients with poor response, evidence‐based strategies in medical failure, management of patients who refuse surgery, the impact of medical treatment on the long‐term disease course and keeping informed about current new treatment options. A. D’Hoore (Belgium) highlighted the fact that, despite medical treatment, a third of patients with acute severe UC will require emergent surgery, and stated that minimally invasive colectomy should be the standard of care, avoiding delay when necessary. P. Myrelid (Sweden) presented all the available options for restoration of bowel continuity, ranging from ileorectal anastomosis, ileoanal pouch, Kock pouch and permanent ileostomy, stating that the ultimate decision should involve surgical expertise and the patient. D. Laharie (France) suggested that nonresponsive patients should be identified as soon as possible, and elderly/fragile patients should probably be offered surgery earlier.

During one of the ‘Big debates’ sessions, ‘Colostomy: mesh it or not’, P. Kirchooff (Switzerland) advocated the use of prophylactic mesh and N. Smart (UK) argued contrary reasons against it. In the second debate ‘Early rectal cancer’, G. Beets (Netherlands) promoted the case for radiotherapy and A. Wolthuis (Belgium) recommended against. A stimulating discussion followed, chaired by E. Espín‐Basany (Spain) and I. Aslam (UK).

O. Zmora (Israel) and G. van Ramshorst (Netherlands) chaired the ‘Trials methodology’ session. P. Hardy (UK) introduced the basics of hypothesis testing, focusing on the importance of asking the right research question. S. Blackwell (UK) presented on involving patients in a trial design. She advocated for patient integration rather than involvement. N. Brindl (Germany) provided an overview of EuroSurg, a student‐led and trainee‐led collaborative that has delivered several successful studies [7, 8], and R. Gujjuri (UK) introduced one of the current projects, the COMPASS study, regarding the use of drains in colorectal surgery [9].

The multidisciplinary session on total adjuvant treatment (TNT) for rectal cancer, chaired by K. Matzel (Germany), was opened with a lecture on the rationale for TNT and initial successes by A. Cervantes (Spain), who highlighted the specific indications and advantages of this approach. C. Van De Velde (Netherlands) showed data from the most recent trials on TNT. R. O. Perez (Brazil) discussed the importance of TNT within the watch and wait pathway. Lastly, J. Hill (UK) discussed microsatellite instability and immunotherapy in this setting. In a concurrent live ESCP online Twitter poll, 58% of 89 respondents felt they would use TNT selectively in rectal cancer.

The session on emergency CRC treatment, chaired by D. Miskovic (UK) and I. Rubio‐Perez (Spain), started with M. Millan (Spain), illustrating the best strategy to manage malignant left‐sided obstruction. J. Stijns (Belgium) talked about the indications for surgery on acute severe UC, showing how to safely perform subtotal colectomy. R. Hompes (Netherlands), showed what to do if a left‐sided anastomotic leakage occurs, highlighting the importance of pro‐active postoperative monitoring with the aim of getting an early diagnosis. The latter will result in a higher chance of preserving the anastomosis. Lastly, A. García‐Granero (Spain) showed tips and tricks to drain perianal abscesses, moving from knowledge of anatomy to intraoperative decisions and provided a useful algorithm to manage each specific patient.

G. Möslein (Germany) chaired the session on ‘Gender and diversity’. M. Penna (UK) gave her perspective on surgeons who are mothers. ‘Parent surgeons’ needed teamwork, not forgetting themselves or their partners, and noted that there are many ways of parenting. S. Markar (UK) (Penna’s husband and also a surgeon), gave the perspective of surgeons who are fathers and on parenting, noting that times are changing. He stated one should use any help available and always find time for rest and relaxation. J. Mayol (Spain) spoke about SoMe in surgery, and how one can build social capital by networking on SoMe. J. Tuynman (Netherlands) presented the role of colorectal surgeons in gender‐affirming surgery focusing on puberty‐suppressing hormones and primary total laparoscopic sigmoid vaginoplasty and its outcomes. F. Marinello (Spain) explored overcoming prejudice and diversity bias in colorectal surgery in a talk in which he also showed the results of a survey that he designed and circulated among colorectal surgeons preceding the talk. He concluded that one should not ‘passively’ stand ‘against’ discrimination or ‘for’ diversity and equality, but that individual surgeons and entities should lead the way. Remarkably in a live audience poll running alongside this presentation more than 50% of 66 respondents had seen or experienced gender and diversity bias in colorectal surgery.

The New Trials Forum on the third day was chaired by T. Pinkney (UK), and started with a presentation of the planned MEErKAT study (Mesenteric Excision and Kono‐S Anastomosis Trial, EME, NIHR131988), presented by S. Brown (UK), which aims to clarify the role of the mesentery and the type of anastomosis on recurrence after ileocaecal resection for CD. The COLOR III trial (NCT02736942), a noninferiority multicentre randomized controlled trial (RCT) comparing transanal total mesorectal excision (TME) versus laparoscopic TME for mid and low rectal cancer, was presented by A. Van Lieshout (Netherlands). M. Mekhael (Denmark) presented a study comparing transanal irrigation versus suppository in patients with major low anterior resection syndrome (LARS) after low anterior resection for rectal cancer. U. Grossi (Italy) presented a phase III, double‐blinded, two‐armed RCT (FIDELIA) on the role of skeletal muscle‐derived cell implantation for the treatment of faecal incontinence. This was followed by T. C. Sluckin (Netherlands) who presented the THROS trial, a RCT to compare rubber band ligation versus sclerotherapy for symptomatic stage 1–2 haemorrhoids. Lastly, G. Van Ramshorst summarized the results of the 2021 ESCP wound closure survey and S. Chaudhri (UK) presented the CORREA 2021 audit, a ESCP snapshot audit on colorectal resections.

In a keynote, chaired by G. Moslein (Germany), T. Poskus (Lithuania) spoke about the centralization of rectal cancer care, showing the different national approaches in relation to rectal cancer treatment and the importance of centralization across Europe in order to achieve better results.

S. Breukink (Netherlands) chaired the “Pregnancy and CRC” session. J. Walsh (Dublin) presented indications for caesarean section and what the colorectal surgeon should know about it. Moreover, she highlighted the matter of peri‐mortem caesarean section, within 5 min of mother's collapse in order to assist maternal resuscitation. M. Collie (UK) talked about postpartum coordination between the obstetrician and the colorectal surgeon. She described the commonest postpartum complications that necessitate a colorectal surgeon involvement and available treatment approaches. C. Selinger (UK) talked about the method of delivery in the patients with inflammatory bowel disease and illustrated the key‐points in this group of patients: multidisciplinary decision‐making during pregnancy, frequent communication between healthcare professionals, and patient involvement.

M. Millan and S. Tou (UK) chaired the ‘Trainee video’ session, in which technical tips of several colorectal index procedures were shown, including how to perform the perfect stoma (I. Rubio‐Perez, Spain), techniques to avoid ureteric injuries (D. Zimmerman, Netherlands), ileocolic anastomosis after right colectomy (J. Stijns, Belgium), shaving and discoid resection for endometriosis (M. Zawadzky, Poland) and Ferguson haemorrhoidectomy with pudendal block (F. Ris, Switzerland).

M. Adamina (Switzerland) chaired the ESCP–European Association for Endoscopic Surgery session. M. Barberio (Italy) showed clinical applications of advanced imaging and artificial intelligence. He talked about fluorescence‐guided and hyperspectral surgery, leading the way to precision surgery. A. Arezzo (Italy) provided an update on surgical robots and future perspectives and how technologies are evolving in the next years. M. Chand (UK) presented time‐tested and innovative ways to prevent anastomotic leakage, focusing on marginal gains.

O. Zmora (Israel) chaired the ESCP–European Cancer Organisation (ECCO) session. C. Buskens (Netherlands) talked about new insights and surgical innovations in management of inflammatory bowel disease (IBD). She focused not only on technical innovations but also on IBD management, pursuing a patient‐tailored approach. M. Adamina (Switzerland), lectured on the recent ECCO guidelines on current management of CD describing important steps on the patient’s pathway according to the guideline statements. O. Faiz (UK) gave an inspiring talk on IBD surgery and his aspiration for a quality revolution. He pointed out that this needs to be evidence‐based, correctly measured and that political will must be maximized through timing. He finished by pointing out that moving towards quality in IBD surgery is ‘evolution’ more than ‘revolution’. The session ended with P. Myrelid (Sweden) discussing the surgical options in the management of UC. He highlighted the current knowledge on appendectomy, diverting ileostomy, segmental resections and ileorectal anastomosis in UC patients.

S. Brown and E. Carrington (UK) chaired the session on pelvic floor surgery. J. Cornish (UK) gave an overview on LARS, ranging from physiotherapy, timing of closure of ileostomy after anterior resection and the role of new therapies such as faecal microbiota transplantation. P. Christensen (Denmark) presented some pearls of wisdom on the diagnosis and treatment of chronic anal pain and how some patients may be treated with botulinum toxin injections. Lastly, C. Knowles (UK) presented nonpharmacological treatments for faecal incontinence.

Continuing the theme, S. Brown and C. Knowles (UK) chaired the session on functional disorders of the pelvic floor. J. Martellucci (Italy) emphasized the importance of the neuroenteric axis. Other talks included an overview on constipation by C. Melchior (Sweden), where some definitions in relation to different types of constipation and treatments were presented, and the functional results obtained after ventral rectopexy by C. Cunningham (UK).

G. Gallo (Italy) and I. Negoi (Romania) chaired the ESCP guidelines update session. S. Breukink (Netherlands) presented the work of the ESCP Guidelines Committee during 2020–2021, as well as ongoing projects. Y. Maeda (UK) gave a comprehensive talk on novel instruments and tools for data analysis and evidence grading, focusing on the updates in methodology using examples from the already produced guidelines. G. Van Ramshorst (Netherlands) presented new guidance on the use of pelvic mesh in colorectal surgery [10]. G. Pellino (Italy) went through the ESCP considerations for resuming normal colorectal services amidst the Covid pandemic [11].

In addition, we thank our industry sponsors who delivered an outstanding plethora of high‐quality educational sessions in addition to our core programme above. Intuitive presented robotic‐assisted indications and results for robotic complete mesocolic excision and intra‐corporeal anastomosis. Ethicon promoted two symposia, the first focused on research to diminish surgical complications, presenting the EAGLE study as an example, and the second focused on the use of new digital technology in colorectal surgery daily practice. Medtronic’s symposium was about fluorescence imaging in colorectal surgery. Takeda focused on the approach to complex Crohn’s perianal fistulas. B. Braun, also organized two symposia, one on minimally invasive techniques in intracorporeal left colectomy and another on endoluminal vacuum therapy to treat anastomotic leakages. WCP addressed the evolution towards dedicated colorectal surgeons: problems, challenges and opportunities. THD presented minimally invasive surgical treatments for haemorrhoids. Ezisurg Medical focused on the association between surgical technical skills and long‐term survival in CRC. CMR Surgical showed how to perform a colectomy in a minimally invasive or robotic fashion.

The conference overall reflected a diverse and enthusiastic faculty and has since provided excellent feedback, to the delight of the Executive and as answer to their efforts, but we could not quite dampen the resolute feeling of sadness at our collective loss of another face‐to‐face opportunity to meet again with friends and network with mentors and collaborators. The entire Executive thanks our speakers, faculty and attendees as well as our extensive conference support network of planners, platforms and technicians. The team are now deep in planning for our next congress, hopefully face‐to‐face, in the beautiful and welcoming city of Dublin in September 2022, for the ESCP’s 17th Annual Scientific Congress.
